# Combining Neuroimaging and Omics Datasets for Disease Classification Using Graph Neural Networks

**DOI:** 10.3389/fnins.2022.866666

**Published:** 2022-05-23

**Authors:** Yi Hao Chan, Conghao Wang, Wei Kwek Soh, Jagath C. Rajapakse

**Affiliations:** School of Computer Science and Engineering, Nanyang Technological University, Singapore, Singapore

**Keywords:** attention, diffusion tensor imaging, disease classification, functional magnetic resonance imaging, Generative Adversarial Networks, graph convolutional networks, multi-omics, Parkinson's disease

## Abstract

Both neuroimaging and genomics datasets are often gathered for the detection of neurodegenerative diseases. Huge dimensionalities of neuroimaging data as well as omics data pose tremendous challenge for methods integrating multiple modalities. There are few existing solutions that can combine both multi-modal imaging and multi-omics datasets to derive neurological insights. We propose a deep neural network architecture that combines both structural and functional connectome data with multi-omics data for disease classification. A graph convolution layer is used to model functional magnetic resonance imaging (fMRI) and diffusion tensor imaging (DTI) data simultaneously to learn compact representations of the connectome. A separate set of graph convolution layers are then used to model multi-omics datasets, expressed in the form of population graphs, and combine them with latent representations of the connectome. An attention mechanism is used to fuse these outputs and provide insights on which omics data contributed most to the model's classification decision. We demonstrate our methods for Parkinson's disease (PD) classification by using datasets from the Parkinson's Progression Markers Initiative (PPMI). PD has been shown to be associated with changes in the human connectome and it is also known to be influenced by genetic factors. We combine DTI and fMRI data with multi-omics data from RNA Expression, Single Nucleotide Polymorphism (SNP), DNA Methylation and non-coding RNA experiments. A Matthew Correlation Coefficient of greater than 0.8 over many combinations of multi-modal imaging data and multi-omics data was achieved with our proposed architecture. To address the paucity of paired multi-modal imaging data and the problem of imbalanced data in the PPMI dataset, we compared the use of oversampling against using CycleGAN on structural and functional connectomes to generate missing imaging modalities. Furthermore, we performed ablation studies that offer insights into the importance of each imaging and omics modality for the prediction of PD. Analysis of the generated attention matrices revealed that DNA Methylation and SNP data were the most important omics modalities out of all the omics datasets considered. Our work motivates further research into imaging genetics and the creation of more multi-modal imaging and multi-omics datasets to study PD and other complex neurodegenerative diseases.

## 1. Introduction

Neurodegenerative diseases such as Parkinson's Disease (PD) have been shown to be associated with both brain connectivity and genetic factors. While measurements of cortical thickness from structural Magnetic Resonance Imaging (MRI) have produced contradictory findings about its utility to predict PD (Yadav et al., 2016), analysis of Diffusion Tensor Imaging (DTI) data has consistently shown that PD patients, with and without cognitive deficits, have reduced fractional anisotropy in prefrontal areas (Deng et al., 2013; Price et al., 2016). Studies on functional MRI (fMRI) data have also consistently revealed lower activity in the supplementary motor complex (Nachev et al., 2008), reduced functional connectivity in the posterior putamen (Herz et al., 2014), as well as changes in the activity levels of the dopaminergic cortico-striatal (Tessitore et al., 2019) and mesolimbic-striatal loops (Filippi et al., 2018) in PD patients.

On the genomics front, several genes (such as alpha-synuclein, LRRK2 and PARK2) and their variants, in the form of Single Nucleotide Polymorphism (SNP) data, have been associated with PD (Klein and Westenberger, 2012). However, none of them have complete penetrance and it is likely that there are multiple risk factors involved in both familial and sporadic PD (Tran et al., 2020), as well as influence from non-coding ribonucleic acid (RNA) (Majidinia et al., 2016). Thus, small non-coding RNA (sncRNA) such as micro RNA (miRNA) should be considered as well. miRNA has been associated with PD: the mitochondrial cascade hypothesis stems from miRNA dysregulation, which causes oxidative stress in neurons and ultimately lead to aggregation of alpha-synuclein and neurodegeneration (Watson et al., 2019). With sporadic PD representing a much larger proportion of PD cases as compared to familial PD, epigenetics alterations (such as DNA Methylation) could be a potential biomarker for PD (Miranda-Morales et al., 2017). Recent findings have revealed that hypo-regulation of some PD-associated genes, such as the SNCA promoter region, upregulates SNCA and leads to the formation of Lewy bodies (Wang et al., 2019).

Neuroimaging and multi-omics data capture different aspects of brain disease manifestations. Neuroimaging modalities such as DTI and fMRI capture macroscopic differences in the structure and function of healthy and diseased brains while multi-omics data zoom into a microscopic view of various molecular signatures in neurodegenerative diseases. Although these modalities have been implicated in PD, their relative importance over each other is less clear. Thus, integrating imaging and omics modalities could reveal new links between these levels of analysis and unravel the pathway of complex neurodegenerative diseases such as PD (Antonelli et al., 2019). However, methods to combine imaging and genetics data are very limited. Existing studies typically study multi-modal imaging data (Subramanian et al., 2020) and multi-omics data (Chaudhary et al., 2018; Zhang et al., 2018; Jin et al., 2021) separately, or combine one imaging modality with only one omics dataset (Kim et al., 2017; Markello et al., 2021). Notably, there have also been works that merged multi-modal imaging data with non-imaging data such as demographic features (Kazi et al., 2019a,b); as well as combining genetic data with clinico-demographic data (Nalls et al., 2015). However, none has attempted to combine both multi-modal imaging and multi-omics data.

One reason for this is due to the very large number of features involved in both imaging and omics datasets. Depending on the choice of atlases, structural and functional connectivity matrices could introduce several thousands of features, while omics datasets are even bigger, ranging from thousands in sncRNA to half a million in DNA Methylation data. Existing methods to combine both data modalities are rudimentary and often involve concatenation. This makes modeling challenging, especially because number of data samples with both imaging and omics data are very few. Models trained on such small datasets overfit easily.

To overcome these issues, we propose a deep neural network architecture that uses a combination of graph convolution layers and the attention mechanism to model multi-modal imaging and multi-omics datasets simultaneously. This is demonstrated on the Parkinson's Progression Markers Initiative (PPMI) dataset, which has a rich collection of imaging (DTI, fMRI) and omics datasets (SNP, sncRNA, miRNA, RNA sequencing and DNA Methylation). However, the number of disease classification studies based on this dataset has been limited, likely due to the very imbalanced distribution of classes (many more PD patients than controls). To alleviate the problem of imbalanced data, we propose the use of CycleGAN to generate structural and functional connectivity matrices of healthy subjects to augment the existing dataset. Existing methods for addressing class imbalance are not feasible for our problem—synthetic data generation algorithms such as SMOTE and ADASYN could generate more data but it will not be possible to associate them to a particular set of omics data sample. Under-sampling exacerbates the issue of having small datasets, while over-sampling merely duplicates the existing dataset. Given a structural connectivity matrix, CycleGAN is able to generate a functional connectivity matrix (and vice versa) such that it corresponds to the same subject and it is not just another repeated data sample in the existing dataset.

With these augmented and less imbalanced datasets, we propose an architecture named JOIN-GCLA (Joining Omics and Imaging Networks *via* Graph Convolutional Layers and Attention) to model both connectome and genomics data simultaneously. Based on our proposed algorithm, a population graph generated from both structural and functional connectivity matrices is used as the graph of the graph convolution layer. Thus, the learnt embedding of the feature vectors—which could be arbitrarily chosen—will be influenced by the multi-modal imaging data. The learnt representations are then passed into multiple graph convolution layers, each based on a graph that is built using different omics datasets. Each graph convolution layer produces its own intermediate representations and interim prediction. These are fused together *via* an attention mechanism, leading to a final decision of the disease classification problem.

Experiment results showed that the best performing model made use of both multi-modal imaging and multi-omics data. Both were crucial for the good performance—model performance fell significantly when only 1 imaging modality, only 1 omics or when no omics dataset were used. Data augmentation was essential for the models to perform well—without it, the extreme imbalance hinders proper model training even with the use of class-weighted cost functions. JOIN-GCLA was shown to outperform existing approaches of multi-modal fusion (Long et al., 2012; Kazi et al., 2019b). Ablation studies demonstrated the importance of the initial graph convolution layer used to learn representations of the connectome data - replacing the graph convolution layer with fully-connected or convolution layers saw significant reduction in model performance. The proposed attention layer was also shown to outperform a self-attention baseline. Furthermore, JOIN-GCLA provides improved model interpretability. With a carefully designed attention mechanism, the resultant attention matrix revealed that out of the omics datasets used, DNA methylation was the most important omics data when predicting that the data sample is a healthy control, while SNP was most important when predicting PD patients.

In sum, we have made the following novel contributions in this work:

- Proposed an architecture, JOIN-GCLA, that is able to incorporate both multi-modal connectome datasets and multi-omics datasets simultaneously.
- JOIN-GCLA provides better model interpretability from the generated attention score matrix—it is able to identify which omics modalities are being focused on when predicting a certain disease class.
- Found that amongst all the multi-omics datasets used, DNA methylation and SNP are the most important omics modalities for PD classification.

## 2. Methods

### 2.1. JOIN-GCLA Architecture

**Figure 1:**
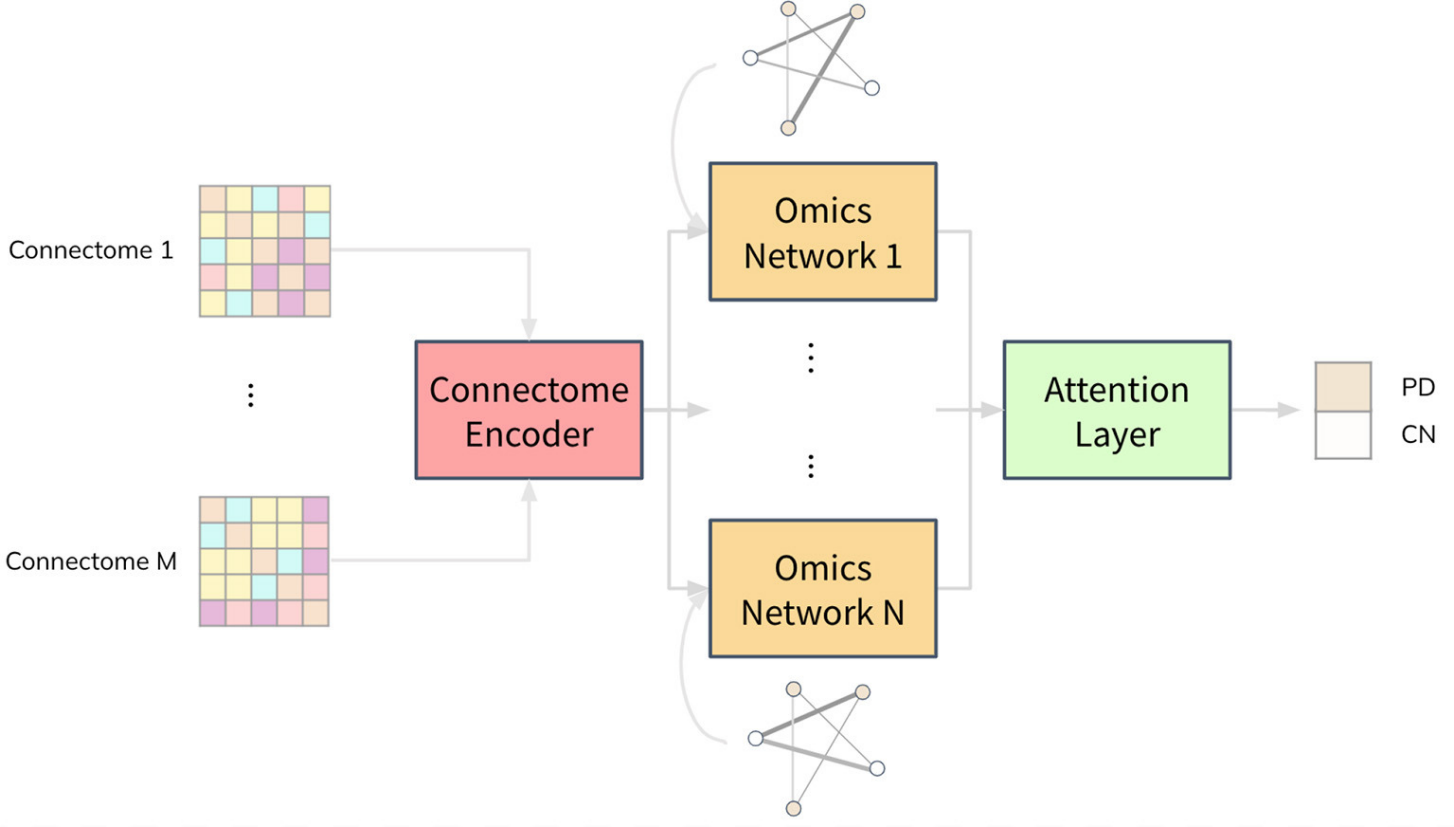
Illustration of the JOIN-GCLA architecture. It is made up of 3 parts: a connectome encoder, omics networks and an attention layer. The connectome encoder receives connectome features from neuroimaging modalities, omics networks embed omics data in their graphs, and the attention layer consolidates all the outputs of the omics networks to make a single final prediction.

We propose a deep neural network architecture, named Joining Omics and Imaging Networks *via* Graph Convolutional Layers and Attention (JOIN-GCLA), that consists of multiple graph convolution layers and an attention mechanism to combine multi-modal imaging data and multi-omics datasets for prediction of PD. Figure 1 illustrates the JOIN-GCLA architecture that is made up of 3 cascaded networks: the connectome encoder, omics networks, and an attention layer.

Fusion of multi-modal imaging data and multi-omics data is performed within the graph convolution layer of the connectome encoder and omics network, respectively. Thus, the inputs to the JOIN-GCLA architecture can be arbitrarily defined, depending on what is desired to be studied. In this work, we use features from the connectomes derived from each imaging modality as inputs to JOIN-GCLA. Let us assume we receive a multi-modal imaging dataset $X={\left\{X^{m}\right\}}_{m=1}^{M}$ with connectivity feature matrix $X^{m}\in {\mathbb{R}}^{P\times J_{m}}$ where *J*_*m*_ is the number of connectivity features derived from each imaging modality *m*, obtained from *P* imaging scans. For the omics networks, the information from *N* omics data types are encoded in the graphs of *N* graph convolution layers. Let $O={\left\{O^{n}\right\}}_{n=1}^{N}$ denote the features of omics data where *N* denotes the number of omics data types and $O^{n}\in {\mathbb{R}}^{P\times K_{n}}$ denotes the features from the *n*-th omics data type. *K*_*n*_ is the number of omics features from each omics data type *n*. Finally, let the set of weights, biases, output and size of the *l*-th layer be denoted by *W*^(*l*)^, *b*^(*l*)^, *H*^(*l*)^ and *L*^(*l*)^, respectively.

#### 2.1.1. Population Graphs

Both the connectome encoder and the omics networks make use of graph convolution layers that decode the information encoded in population graphs where each node in a population graph represents a data sample. The connectome encoder condenses the structural and functional connectivity matrices into a small and compact vector representation. The omics networks receive the representation realized from imaging data and combine them with omics data for disease classification.

The graph of the connectome encoder is built from multiple connectome datasets derived from neuroimaging data. Formally, we define the imaging-based population graph as a population scan graph (PSG) where imaging scans are represented as nodes and the similarity between each pair of scans is calculated as the edge weight, making it a fully connected weighted graph.

Let us denote $x_{v}^{m}$ as the connectivity features for imaging modality *m* from an individual *v* and let $A^{m}=\left\{a_{uv}^{m}\right\}\in {\mathbb{R}}^{P\times P}$ denote the adjacency matrix of a PSG where *u* and *v* denote two data samples. Each weight *a*_*uv*_ represents the similarity sim between two samples:


(1) $$\begin{array}{l} a_{uv}^{m}=\text{sim}\left(x_{u}^{m},x_{v}^{m}\right) \end{array}$$

Similarly, we make population omics graphs (POG) from features of each omics data type. Let $o_{v}^{n}$ represent the omics features for omics data type *n* from an individual *v* and let $B^{n}=\left\{b_{uv}^{n}\right\}\in {\mathbb{R}}^{P\times P}$ represent the adjacency matrix of a POG. Each weight *b*_*uv*_ represents the similarity sim between two samples:


(2) $$\begin{array}{l} b_{uv}^{n}=\text{sim}\left(o_{u}^{n},o_{v}^{n}\right) \end{array}$$

The similarity measure sim is chosen as the Pearson's correlation coefficient.

#### 2.1.2. Connectome Encoder

The connectome encoder is made up of a linear layer and a graph convolution layer. The input to the connectome encoder is the modality-wise concatenation of connectivity feature matrices, represented by *X*^*c*^∈ℝ^*P*×*J*^, where $J=\overset{M}{\underset{m=1}{\sum }}J_{m}$. A linear layer is first used to reduce data dimensionality. This is needed because the connectivity matrices were built by computing correlations between time-series from brain regions-of-interests (ROI), which produces a large number of features. In this work, since both fMRI and DTI data were involved, we used the AAL atlas which defines 116 ROIs and produces 6,670 features for each imaging modality, warranting the need for the linear layer:


(3) $$\begin{array}{l} H^{\left(1\right)}=\text{ReLU}\left(X^{c}W^{\left(1\right)}+b^{\left(1\right)}\right) \end{array}$$

where ReLU denotes the ReLU activation function.

The output of the linear layer is then passed to the graph convolution layer. Additionally, the graph convolution layer takes in a PSG as the graph *A*. The PSG was created by setting the edge weights between each pair of subjects as the Pearson's correlation of their vectorised connectivity matrices. Min-max normalization is then performed on the PSG and each element in the PSG is incremented by 1 to ensure that the minimum value is 1. When there are multiple modalities involved, let the PSG of modality *m* be denoted by *A*^*m*^. *A*^*m*^ is multiplied with the existing PSG *A*, which is initialized as a matrix of ones. *A* is then used as the graph of the graph convolution layer.

Since the PSG is fully connected, the graph convolution layer should incorporate edge weights from the graph when improving the feature vector. One such layer was proposed in Kipf and Welling (2016):


(4) $$\begin{array}{l} H^{\left(2\right)}=\text{ReLU}\left({\hat{D}}_{A}^{-1/2}Â{\hat{D}}_{A}^{-1/2}H^{\left(1\right)}W^{\left(2\right)}\right) \end{array}$$

where Â = *A*+*I* represents the PSG (of dimensions *P*×*P*) with self-loops added, and ${\hat{D}}_{A}=\left\{{\hat{d}}_{vv}\right\}$ represents the diagonal degree matrix of *A* with ${\hat{d}}_{vv}=\underset{u\in V}{\sum }{\hat{d}}_{vu}$ where *V* is the vertex set of scans. The output of the connectome encoder, *H*^(2)^, is subsequently used as input to each omics network.

#### 2.1.3. Omics Networks

Each omics network is made up of a graph convolution layer and a softmax layer. Despite receiving the same output from the connectome encoder, each omics network produces different outputs because the POG used in each omics network is different. Creating the POG *O* involves a different procedure from Parisot et al. (2018) due to the nature of omics datasets. For example, the population graph of DNA Methylation and miRNA data have values very close to each other (as seen in **Figure 3**), which requires further scaling. This is done *via* the WGCNA algorithm (Zhang and Horvath, 2005) which re-scales the values to follow a power law distribution. Furthermore, while one subject has only one set of multi-omics data, a single subject can have multiple imaging scans. Thus, a duplication step has to be introduced to replicate the omics features when a subject has multiple imaging scans.

In short, POGs are generated by producing an adjacency matrix *via* computing the correlation between each scan's omics vector, followed by addition of self-loops, WGCNA scaling and scan duplication for subjects with more than 1 imaging scan, producing an *P*×*P* matrix. Since POGs are also always fully connected, the model proposed in Kipf and Welling (2016) can be used.


(5) $$\begin{array}{l} H^{\left(3\right)}={\hat{D}}_{B}^{-1/2}\hat{B}{\hat{D}}_{B}^{-1/2}H^{\left(2\right)}W^{\left(3\right)} \end{array}$$

where $\hat{B}=B+I$ represents the POG with self-loops added, ${\hat{D}}_{B}=\left\{{\hat{d}}_{vv}\right\}$ represents the diagonal degree matrix of *B*. Subsequently, the output of the graph convolution layer is passed to a linear layer with *L*^(4)^ hidden nodes, where *L*^(4)^ represents the number of classes for the classification task.


(6) $$\begin{array}{l} H^{\left(4\right)}=\text{ReLU}\left(\text{ReLU}\left(H^{\left(3\right)}\right)W^{\left(4\right)}+b^{\left(4\right)}\right) \end{array}$$

The above equations detail the process of generating the outputs of a single omics network. In the case where only a single omics data is available, *H*^(4)^ can be passed to a softmax layer to produce the final prediction. Given *N* different sets of omics data, we will repeat these steps for each omics dataset, each producing their own omics network. Then, both *H*^(3)^ and *H*^(4)^ will be used by the attention layer shown in the next section.

#### 2.1.4. Attention Layer

When multiple omics datasets are used, not all of them will be useful for the classification task. Thus, we introduce an attention layer that learns which omics network to pay more attention to when making the final prediction. For each data sample, the attention layer will learn an attention matrix of dimensions *N*×*L*^(4)^, showing which omics network is being focused on for the classification task. It will also produce a single prediction for the disease classification task.

The attention mechanism, following the terminology in Vaswani et al. (2017), involves two components: (i) the attention weights produced from a pair of query and key matrices, and (ii) the value matrix, i.e. the term to be weighted. The latter refers to *H*^(4)^, the logits from each omics network. Thus, let *H*^(4*c*)^∈ℝ^*P*×*N*×*L*^^(4)^ be the concatenated logits from all omics networks. For the former, since it is desirable to arrive at an attention matrix of dimension *N*×*L*^(4)^ for better model interpretability, the query matrix is defined as *H*^(4*m*)^∈ℝ^*P*×*L*^^(4)^×1, the mean of logits from all omics networks, averaged across dimension *N* and transposed so that the shape of the attention matrix is correct. The key matrix is defined as *H*^(3*c*)^∈ℝ^*P*×*N*×*L*^^(3)^, which represents the combined outputs concatenated from the graph convolution layer in each omics network. Since the last dimension of *H*^(4*m*)^, *H*^(3*c*)^ and *H*^(4*c*)^ are different, *H*^(3*c*)^ is projected *via* a projection matrix *W*^(3*c*)^∈ℝ^*L*^^(3)^×1. Similarly, *H*^(4*c*)^ is projected by *W*^(4*c*)^∈ℝ^*L*^^(4)^×1.

Finally, the query matrix *H*^(4*m*)^ and the key matrix *H*^(3*c*)^ are combined to compute the attention score used to weigh the value matrix *H*^(4*c*)^. In sum, this operation finds the best set of weights to weigh the output of each *H*^(4)^ from the omics networks, producing *H*^(5)^∈ℝ^*P*×^*L*^^(4)^×1^.


(7) $$H^{\left(5\right)}=\text{softmax}\left(H^{\left(4m\right)}{\left(H^{\left(3c\right)}W^{\left(3c\right)}\right)}^{T})(H^{\left(4c\right)}W^{\left(4c\right)})\right)$$

#### 2.1.5. Output Layer

*H*^(5)^ is then passed into a softmax layer to produce the predicted class label *y*.


(8) $$\begin{array}{l} p\left(y_{i}=y_{k}|H^{\left(5\right)}\right)=\text{softmax}\left(H^{\left(5\right)}\right) \end{array}$$

#### 2.1.6. Training

Training of the JOIN-GCLA architecture is done by minimizing the error between predicted class label *y* and the target class label *y*_*d*_ *via* a weighted cross-entropy cost function *J* to account for data imbalance. Let $w_{y_{d}}=1-\frac{P_{y_{d}}}{P}$ be the weight of the class *y*_*d*_, where *P*_*y*_*d*__ refers to the data subset that belongs to the class *y*_*d*_.


(9) $$\begin{array}{l} J=E_{x}\left\{-w_{y_{d}}y_{d}log\left(y\right)-\left(1-w_{y_{d}}\right)\left(1-y_{d}\right)log\left(1-y\right)\right\} \end{array}$$

The cost function *J* is minimized using an Adam optimiser. Also, during model training, dropouts are added after the graph convolution layer in both the connectome encoder and the omics networks.

## 3. Results

### 3.1. Dataset and Pre-processing

Data used in this study were obtained from the Parkinson's Progressive Markers Initiative (PPMI) (Marek et al., 2018). PPMI is a clinical study that seeks to build data driven approaches for early diagnosis of PD by discovering novel biomarkers. For this study, we have utilized both imaging and genetic data downloaded from the website. Tables 1, 2 summarizes key demographic information and statistics of the PPMI dataset for imaging data, while Table 3 shows the sample and feature sizes of the omics datasets. PD subjects included in this study are those who either have a pathogenic genetic variant or are newly diagnosed and have yet to commence medication for PD.

**Table 1:** Basic statistics of subjects with DTI scans in PPMI dataset.

	**Healthy control (HC)**	**Parkinson's disease (PD)**
Number of subjects (scans)	66 (178)	154 (705)
Male/Female	43/23	98/56
Age	60.9 ± 10.6	60.8 ± 9.3

**Table 2:** Basic statistics of subjects with fMRI scans in PPMI dataset.

	**Healthy control (HC)**	**Parkinson's disease (PD)**
Number of subjects (scans)	18 (19)	94 (194)
Male/Female	14/4	64/30
Age	61.0 ± 10.8	59.7 ± 10.2

**Table 3:** Dataset and feature sizes of multi-omics data before and after pre-processing.

**Omics data type**	**Dataset size**	**Original**	**Processed**
		**feature size**	**feature size**
RNAseq	226	34,569	19,728
Met	152	864,067	677,506
SNP	206	267,607	239,731
sncRNA	184	29,585	4,366
miRNA	184	2,656	748

Details about the pre-processing steps are shown in the Supplementary Materials. In brief, after pre-processing the raw diffusion weighted imaging data to correct for motion, eddy currents and echo planar imaging distortions *via* the dwi-preprocessing-using-t1 pipeline in Clinica (Routier et al., 2021), structural connectivity matrices were obtained by performing probabilistic tractography using the BedpostX GPU (Hernández et al., 2013) and ProbtrackX GPU (Hernandez-Fernandez et al., 2019) tool from FSL (Jenkinson et al., 2012). Since the raw connectivity matrix is not symmetric, the average of the upper and lower triangular was computed and was further log-transformed and standardized to ensure that the values follow a standard normal distribution (which will aid downstream modeling tasks). The fMRI dataset was processed using fMRIPrep (Esteban et al., 2019) and the AAL atlas was used to generate 116 regions of interests (ROI) from both the cortex and subcortex. The activation of a ROI is computed by taking the mean time series of all voxels less than 2.5 mm away from the ROI. Pearson correlation was used to obtain a symmetric matrix containing the functional connectivities between pairs of ROIs for each scan.

Most of the DTI and fMRI scans in the PPMI datasets are taken on different sessions (i.e. different days). Just relying on scans which are taken on the same day will result in a small and unusable dataset. Instead, for every DTI scan, we pair it up with fMRI scans which are taken not more than 1 year away from the date the DTI scan was performed. This produces 351 PD and 25 HC scans with paired DTI and fMRI data.

For multi-omics datasets, PPMI provides pre-processed data, with steps such as quality control and normalization performed. RNA-Seq data are given in format of Transcripts Per Million, and sncRNA and miRNA data are given in Reads Per Million (RPM) and RPM Mapped to miRNA formats. DNA Methylation (Met) and Single Nucleotide Polymorphism (SNP) data have been distilled with *p*-value detection. Based on the above processing, we further perform noise removal and Wilcoxon Signed Rank test to eliminate irrelevant features on the sample set required for downstream experiments. More details about the pre-processing steps can be found in the Supplementary Materials.

### 3.2. Data Augmentation

Most multi-modal imaging and multi-omics datasets are small because not all the subjects with one imaging modality come along with other modalities. For instance, not all subjects with DTI scans will have a corresponding fMRI scans (and vice versa). This is also true for the PPMI dataset. Another major issue in the PPMI dataset is the huge class imbalance, with the number of PD subjects about 10 times larger than the number of healthy controls, as seen in Tables 1, 2. To address these issues, we use CycleGAN, a type of Generative Adversarial Network (GAN) proposed by Zhu et al. (2017), to generate functional connectomes from structural connectomes of healthy subjects. GANs are generative models that can generate additional data samples with distributions similar to that of the distribution of the training dataset. CycleGAN is made up of conditional GANs, which are able to use images of one modality as latent variable so as to generate images of another modality. CycleGAN goes further to introduce a cycle consistency loss that ensures that the source and target images are consistent with each other as the network is able to both generate the target image from the source image and reconstruct the source image from the generated target image.

To train the CycleGAN architecture, functional and structural connectivity matrices, generated from preprocessed fMRI and DTI data from the Human Connectome Project (HCP) S1200 release (Glasser et al., 2013), was used as the training data and the CycleGAN model was tuned and tested using data from the Amsterdam Open MRI Collection (AOMIC) (Snoek et al., 2021). PIOP1 was used as validation set, while PIOP2 was the test set. Both HCP and AOMIC datasets are made up of brain imaging scans from healthy young adults. These were chosen, despite the age differences from PPMI, due to the large dataset sizes available (1062 for HCP, 189 for PIOP1 and 183 for PIOP2). To the best of our knowledge, no publicly available datasets with such dataset sizes exist for elderly populations. Pre-processing steps for the HCP and AOMIC datasets are similar to Section 3.1 and more details about the dataset and pre-processing steps are provided in the Supplementary Materials. With a trained CycleGAN model, structural connectivity matrices are passed into it to generate additional fMRI scans of healthy subjects. These are used to augment the original dataset. This results in 208 PD and 186 HC scans, a more balanced dataset (52.3% as compared to 91.6% previously). For the paired DTI-fMRI dataset, this results in 351 PD and 364 HC scans, also resulting in a more balanced dataset (53.3% as compared to 93.4%).

### 3.3. Hyperparameter Tuning

The huge number of possible omics and imaging data combinations makes it unfeasible to tune the model for each of them. Rather, hyperparameter tuning was performed once on the largest dataset available for the baseline model (i.e. a graph convolutional layer, without the omics networks, trained only on DTI data). We first split the dataset into non-test and test sets at a 2:1 ratio, before performing 5 fold cross-validation on the non-test split. Once the optimal parameters are found, the experiments are repeated over 10 seeds and the mean accuracies (along with standard deviation) are reported in the next sections. Importantly, synthetic data are only added to the training set–the validation and test set always uses real data only.

Parameters tuned include dropout {0.1, 0.3, 0.5}, number of hidden neurons in the graph convolution layers {2, 4, 8, 16, 32} and learning rate {0.001, 0.0005, 0.0001}. Early stopping with a patience of 20 epochs was applied during the tuning process and the largest number of epochs taken to reach the best Matthew Correlation Coefficient (MCC) score was used as the number of epochs to train the model for before applying the model on the test set. The optimal parameters are dropout of 0.1, 16 hidden neurons and learning rate of 0.001. Adam optimiser was used to train the model. This set of parameters is consistently used throughout all combinations of data modalities, with no further model tuning done for the other imaging and omics combinations. All experiments were repeated over 10 seeds.

### 3.4. Data Augmentation Improves Disease Classification

The PPMI dataset is heavily imbalanced. Even when the cost function is weighted by the classes, Table 4 showed that the trained JOIN-GCLA model cannot classify well without data augmentation. While the accuracy achieved is high, that is an indication that the model is stuck at predicting the majority class (PD) and cannot predict the minority class (HC) well. Supplementary Table S1 shows the percentage of the dataset represented by the majority class. It is evident that model performance on the original dataset is often around or even below this percentage. Additional confirmation is provided by the MCC scores, which are very low without data augmentation. With data augmentation, MCC increased significantly on most omics combinations. Thus, data augmentation helps to reduce the imbalance and it is necessary for good model performance. Analyses in subsequent sections will use this augmented dataset.

**Table 4:** Comparison of model performance on DTI-fMRI data, with and without training set augmentation.

	**No augmentation**		**With augmentation**	
**Omics**	**Accuracy**	**MCC**	**Accuracy**	**MCC**
None	93.09 ± 0.03	0.00 ± 0.00	93.09 ± 0.03	0.00 ± 0.00
Met	89.59 ± 0.04	0.02 ± 0.05	90.21 ± 0.04	0.13 ± 0.25
SNP	93.18 ± 0.03	0.03 ± 0.09	93.89 ± 0.03	0.08 ± 0.25
miRNA	96.16 ± 0.00	0.00 ± 0.00	96.16 ± 0.00	0.00 ± 0.00
sncRNA	96.16 ± 0.00	0.01 ± 0.01	96.16 ± 0.00	0.00 ± 0.00
RNAseq	92.82 ± 0.03	0.00 ± 0.00	92.82 ± 0.03	0.01 ± 0.04
RNAseq-Met	81.21 ± 0.20	0.17 ± 0.33	92.52 ± 0.10	0.79 ± 0.23
RNAseq-SNP	88.96 ± 0.11	0.28 ± 0.19	84.92 ± 0.13	0.38 ± 0.29
RNAseq-miRNA	87.48 ± 0.26	0.03 ± 0.05	95.65 ± 0.02	0.02 ± 0.03
RNAseq-sncRNA	92.11 ± 0.11	0.02 ± 0.05	96.10 ± 0.01	0.08 ± 0.18
Met-SNP	85.59 ± 0.14	0.39 ± 0.34	83.99 ± 0.13	0.43 ± 0.35
Met-miRNA	90.62 ± 0.20	0.03 ± 0.05	95.72 ± 0.11	0.61 ± 0.51
Met-sncRNA	85.99 ± 0.24	0.02 ± 0.05	98.16 ± 0.02	0.43 ± 0.49
SNP-miRNA	96.84 ± 0.03	0.04 ± 0.10	100.0 ± 0.00	1.00 ± 0.00
SNP-sncRNA	85.91 ± 0.29	0.02 ± 0.06	99.78 ± 0.01	0.90 ± 0.32
miRNA-sncRNA	94.87 ± 0.04	0.06 ± 0.15	96.25 ± 0.01	0.06 ± 0.18
RNAseq-Met-SNP	89.45 ± 0.04	0.37 ± 0.25	86.46 ± 0.16	0.56 ± 0.38
RNAseq-Met-miRNA	97.13 ± 0.01	0.01 ± 0.01	97.93 ± 0.01	0.29 ± 0.45
RNAseq-Met-sncRNA	97.28 ± 0.01	0.11 ± 0.25	97.80 ± 0.02	0.32 ± 0.47
RNAseq-SNP-miRNA	88.64 ± 0.30	0.01 ± 0.02	99.63 ± 0.01	0.81 ± 0.40
RNAseq-SNP-sncRNA	97.99 ± 0.00	0.02 ± 0.03	99.77 ± 0.01	0.90 ± 0.30
RNAseq-miRNA-sncRNA	90.18 ± 0.19	0.04 ± 0.06	95.60 ± 0.02	0.01 ± 0.03
Met-SNP-miRNA	96.62 ± 0.01	0.06 ± 0.20	99.68 ± 0.01	0.91 ± 0.30
Met-SNP-sncRNA	96.94 ± 0.01	0.16 ± 0.33	100.0 ± 0.00	1.00 ± 0.00
Met-miRNA-sncRNA	90.23 ± 0.22	0.00 ± 0.02	98.70 ± 0.01	0.52 ± 0.50
SNP-miRNA-sncRNA	90.77 ± 0.23	0.01 ± 0.01	99.80 ± 0.01	0.90 ± 0.32
RNAseq-Met-SNP-miRNA	87.20 ± 0.29	0.12 ± 0.31	99.72 ± 0.01	0.90 ± 0.32
RNAseq-Met-SNP-sncRNA	85.23 ± 0.29	0.05 ± 0.10	99.42 ± 0.01	0.80 ± 0.42
RNAseq-Met-miRNA-sncRNA	96.70 ± 0.01	0.03 ± 0.06	97.08 ± 0.03	0.31 ± 0.48
RNAseq-SNP-miRNA-sncRNA	98.16 ± 0.01	0.11 ± 0.31	99.36 ± 0.01	0.70 ± 0.48
Met-SNP-miRNA-sncRNA	87.39 ± 0.29	0.08 ± 0.17	93.21 ± 0.21	0.91 ± 0.29
RNAseq-Met-SNP-miRNA-sncRNA	96.78 ± 0.01	0.19 ± 0.31	89.67 ± 0.30	0.73 ± 0.44

### 3.5. Effects of Incorporating Different Omics Datasets

JOIN-GCLA takes in two or more omics networks. When less than two omics datasets are available, the attention layer can be removed. Thus, in the case where one omics dataset is used, the resulting architecture has 2 graph convolution layers (1 for imaging, 1 for omics). When no omics datasets are used, the resulting architecture has 1 graph convolution layer for the multi-modal imaging data only. From Table 4, it is evident that almost all the models trained without omics data or only with a single omics data modality fared poorly, with MCC ranging from 0.00 to 0.13 as compared to the multi-omics models (bolded rows) with MCC ranging from 0.73 to 1.00. Furthermore, it is observed that data augmentation has greatest efficacy when multi-omics data is involved. The increase of MCC score ranged from 0.00 to 0.11 when no or one omics data was used, while the increment for multi-omics combinations ranged from 0.54 to 0.84.

### 3.6. Selection of the Optimal Omics Combination

In Table 4, results for the power set of omics combinations were shown for completeness. A principled way to arrive at the optimal combination of omics data is to perform backward elimination at the level of omics data type, based on MCC score. From the full set of omics data (RNAseq-Met-SNP-miRNA-sncRNA), *m*−1 separate models are trained independently, each with a different subset of *m*−1 omics data types obtained by removing a different omics dataset for each model. If any of the new models produces a higher MCC score than the existing best model (initialized as the original set), it is set as the best model and the process continues recursively until it gets terminated when either no omics data is left or the current iteration of models do not perform better than the existing best model from the previous iteration. Following this procedure, Met-SNP-sncRNA was determined to be the optimal omics combination. For clearer presentation of results, subsequent analyses will focus on the rows in bold in Table 4, which represent the best models for each number of omics combinations considered in the process of backward elimination. We adopt the following notation in the tables below: Model 3 = Met-SNP-sncRNA, Model 4 = Met-SNP-miRNA-sncRNA, Model 5 = RNAseq-Met-SNP-miRNA-sncRNA.

### 3.7. Effect of Using Multi-Modal Imaging Data

Table 5 shows that models using multi-modal imaging data generally results in better MCC score than models trained with uni-modal imaging data^1^. In particular, Met-SNP-sncRNA is able to achieve a MCC score of 1 across all 10 seeds, but when DTI data was dropped, the MCC score reduced to 0.80 (p-value of 0.04 when performing a *t*-test to check for identical population means). Higher MCC score was also observed for Met-SNP-miRNA-sncRNA when multi-modal imaging data was involved. While the accuracies obtained when only fMRI used seems generally higher, their lower MCC suggest that the model still tends to predict the majority class. This issue is alleviated when multi-modal imaging data are used.

**Table 5:** Comparison of model performance between DTI-fMRI data and fMRI data.

	**DTI-fMRI**		**fMRI**	
**Omics**	**Accuracy**	**MCC**	**Accuracy**	**MCC**
Model 3	100.0 ± 0.00	1.00 ± 0.00	97.15 ± 0.03	0.80 ± 0.27
Model 4	93.21 ± 0.21	0.91 ± 0.29	96.16 ± 0.04	0.71 ± 0.34
Model 5	89.67 ± 0.30	0.73 ± 0.44	97.43 ± 0.04	0.77 ± 0.41

### 3.8. JOIN-GCLA Outperforms Existing Approaches for Disease Classification

To the best of our knowledge, there has been no existing work proposed to process both multi-modal imaging and multi-omics data in a single architecture. Early methods such as Long et al. (2012) extracted features from structural and functional brain images and used a support vector machine (SVM) to perform disease classification. However, such approaches do not combine omics features. Nevertheless, a comparison will be made between JOIN-GCLA and machine learning models such as SVM and logistic regression (LR) to ascertain whether JOIN-GCLA give any advantage over these models.

Tuning of the machine learning models was performed with Optuna (Akiba et al., 2019) and the models were implemented in Python using Scikit-learn. For SVM, a linear SVM was used and the regularization parameter C is randomly sampled from a log uniform distribution ranging between 1 × 10^−5^ and 1 × 10^5^. For LR, besides the regularization parameter C (sampled from 1 × 10^−3^ to 1 × 10^2^), the parameter l1_ratio is sampled from a uniform distribution ranging between 0 and 1. The best set of model parameters across 10 trials are used to train the final model. Model performance over 10 seeds is reported in Table 6.

**Table 6:** Comparison between alternative fusion approaches and JOIN-GCLA.

**Model**	**Modality**	**Accuracy**	**MCC**
Logistic Regression	DTI	45.07 ± 5.26	–0.10 ± 0.11
Logistic Regression	fMRI	56.84 ± 3.74	0.20 ± 0.07
Logistic Regression	DTI + fMRI	58.53 ± 4.96	0.22 ± 0.10
Support Vector Machine	DTI	46.47 ± 4.96	–0.06 ± 0.12
Support Vector Machine	fMRI	45.87 ± 4.73	0.16 ± 0.11
Support Vector Machine	DTI + fMRI	37.05 ± 8.56	0.14 ± 0.10
JOIN-GCLA, Model 3	DTI + fMRI	100.0 ± 0.00	1.00 ± 0.00
JOIN-GCLA, Model 4	DTI + fMRI	93.21 ± 0.21	0.91 ± 0.29
JOIN-GCLA, Model 5	DTI + fMRI	89.67 ± 0.30	0.73 ± 0.44

While it is evident that the JOIN-GCLA results with multi-omics data outperforms machine learning models, comparing the results in Table 6 with the rows in Table 4 where no omics datasets were used, deep learning models do not seem to perform better than SVM nor logistic regression models. This is true for both cases where fMRI or DTI-fMRI datasets are used. This suggest that the good model performances seen in Table 4 are likely contributed by the addition of omics dataset and the omics networks, rather than just the use of deep learning models in the connectome encoder. While the number of test samples involved in these 3 examples (~55) are indeed smaller than the number of test samples used when no omics data are involved (~115), the difference in performance is unlikely to be attributed to the difference in sample sizes between the experiments. This is supported by the result from omics combination RNAseq-SNP-miRNA-sncRNA, which still has an MCC score of 0.70 with ~95 test samples, much higher than what was obtained from machine learning models despite having a similar number of test samples.

More recent works related to JOIN-GCLA include architectures that combine both imaging data and demographic information in the form of population graphs (Parisot et al., 2018; Kazi et al., 2019a). However, they do not use omics datasets. The closest architecture to JOIN-GCLA is the multi-layered parallel graph convolutional network presented in Kazi et al. (2019a). In their model, separate population graphs were built based on each demographic feature used (e.g. age, gender). Each population graph was used as the graph for a different graph convolutional network (GCN). Features from MRI, fMRI and cognitive tests were used as the node vector of the GCNs. The representations learnt by the GCNs were then fused *via* a weighted sum, with the weight assigned to each GCN being a parameter learnt during model training. JOIN-GCLA is different in two key aspects: (i) our connectome encoder can incorporate multiple modalities of connectome data and (ii) our proposed attention layer is used for fusing multiple views of information. In our implementation of Kazi et al. (2019a), instead of using demographic information, POGs were used as the graph for the graph convolution layers and the connectome encoder is replaced by a fully-connected layer. Table 7 shows that JOIN-GCLA significantly outperforms their approach of modality fusion.

**Table 7:** Comparison between JOIN-GCLA with alternative fusion methods.

	**JOIN-GCLA**		**Kazi et al. (** **2019a** **)**	
**Omics**	**Accuracy**	**MCC**	**Accuracy**	**MCC**
Model 3	100.0 ± 0.00	1.00 ± 0.00	73.71 ± 0.22	0.32 ± 0.32
Model 4	93.21 ± 0.21	0.91 ± 0.29	82.14 ± 0.17	0.18 ± 0.18
Model 5	89.67 ± 0.30	0.73 ± 0.44	77.11 ± 0.19	0.35 ± 0.27

### 3.9. Effects of Graph Convolution Layer in the Connectome Encoder

The connectome encoder in JOIN-GCLA can also be compared with other deep learning approaches by replacing the graph convolution layer with alternatives such as layers in the connectome convolutional neural network proposed by Meszlényi et al. (2017), which uses customized horizontal and vertical filters of dimensions 1 × |*ROI*| and |*ROI*| × 1, respectively. Such a model can accept multi-modal imaging data by treating each modality as an additional channel. Alternatively, the graph convolution layer could be simply replaced with a linear layer. Such a model will take in multi-modal imaging data by flattening the original matrices into vectors and concatenating them into one large feature vector.

From Table 8, it can be seen that both models with the fully-connected layer and convolution layers perform rather poorly. The connectome convolution layers does not seem to aid model performance relative to the fully connected layers. Both model performances are also inferior to the results obtained by JOIN-GCLA, as shown in Figure 1. A limitation of the comparison made in Table 8 is the significantly smaller number of parameters involved in the model with the convolution layers (~30, 000) as compared to the model with the fully-connected layer and JOIN-GCLA (~200, 000). In view of this, another experiment was performed where the number of parameters in the model with convolution layers was increased by increasing the number of filters (for the convolution layer in connectome encoder) and hidden nodes (for the graph convolution layer in omics networks) such that the total number of parameters is similar to the other two models. Results shown in Supplementary Table S5 demonstrates that the larger model using convolution layers is still outperformed by JOIN-GCLA. Thus, it is evident that the proposed method to fuse multi-modal imaging data *via* PSG helps to improve model performance.

**Table 8:** Ablation study of the connectome encoder on DTI-fMRI dataset.

	**JOIN-GCLA**		**Fully-connected layer**		**Convolution layers**	
**Omics**	**Accuracy**	**MCC**	**Accuracy**	**MCC**	**Accuracy**	**MCC**
Model 3	100.0 ± 0.00	1.00 ± 0.00	85.82 ± 0.11	0.42 ± 0.27	95.59 ± 0.09	0.57 ± 0.46
Model 4	93.21 ± 0.21	0.91 ± 0.29	83.69 ± 0.20	0.47 ± 0.39	88.62 ± 0.23	0.26 ± 0.34
Model 5	89.67 ± 0.30	0.73 ± 0.44	89.97 ± 0.11	0.54 ± 0.36	72.86 ± 0.32	0.23 ± 0.30

### 3.10. Effects of Different Attention Layers for Fusing Multi-View Data

Section 3.5 demonstrated the importance of using multi-omics datasets and showed how the attention mechanism improves the final disease prediction. In this section, this will be compared with alternative approaches to fuse the representations learnt from each omics network. One baseline for comparison is to use self-attention, instead of the customized formulation of the attention mechanism proposed in Section 2.1.4. Table 9 shows that our proposed attention layer performs better than self-attention.

**Table 9:** Ablation study of the attention layer on DTI-fMRI dataset.

	**JOIN-GCLA**		**Self-attention**	
**Omics**	**Accuracy**	**MCC**	**Accuracy**	**MCC**
Model 3	100.0 ± 0.00	1.00 ± 0.00	99.31 ± 0.01	0.80 ± 0.42
Model 4	93.21 ± 0.21	0.91 ± 0.29	98.94 ± 0.02	0.70 ± 0.48
Model 5	89.67 ± 0.30	0.73 ± 0.44	98.46 ± 0.02	0.62 ± 0.49

### 3.11. Model Interpretability

The performance of models with graph convolution layers is highly dependent on the graph used (Parisot et al., 2018; Cosmo et al., 2020). This warrants the need to analyse the PSG used in the connectome encoder and POGs used in the omics networks. Additionally, our proposed method to construct the attention scores allows for greater interpretability into the models decision from the weights assigned to the intermediate representations produced from the omics networks when predicting HC or PD.

#### 3.11.1. Imaging Population Scan Network Distributions

**Figure 2:**
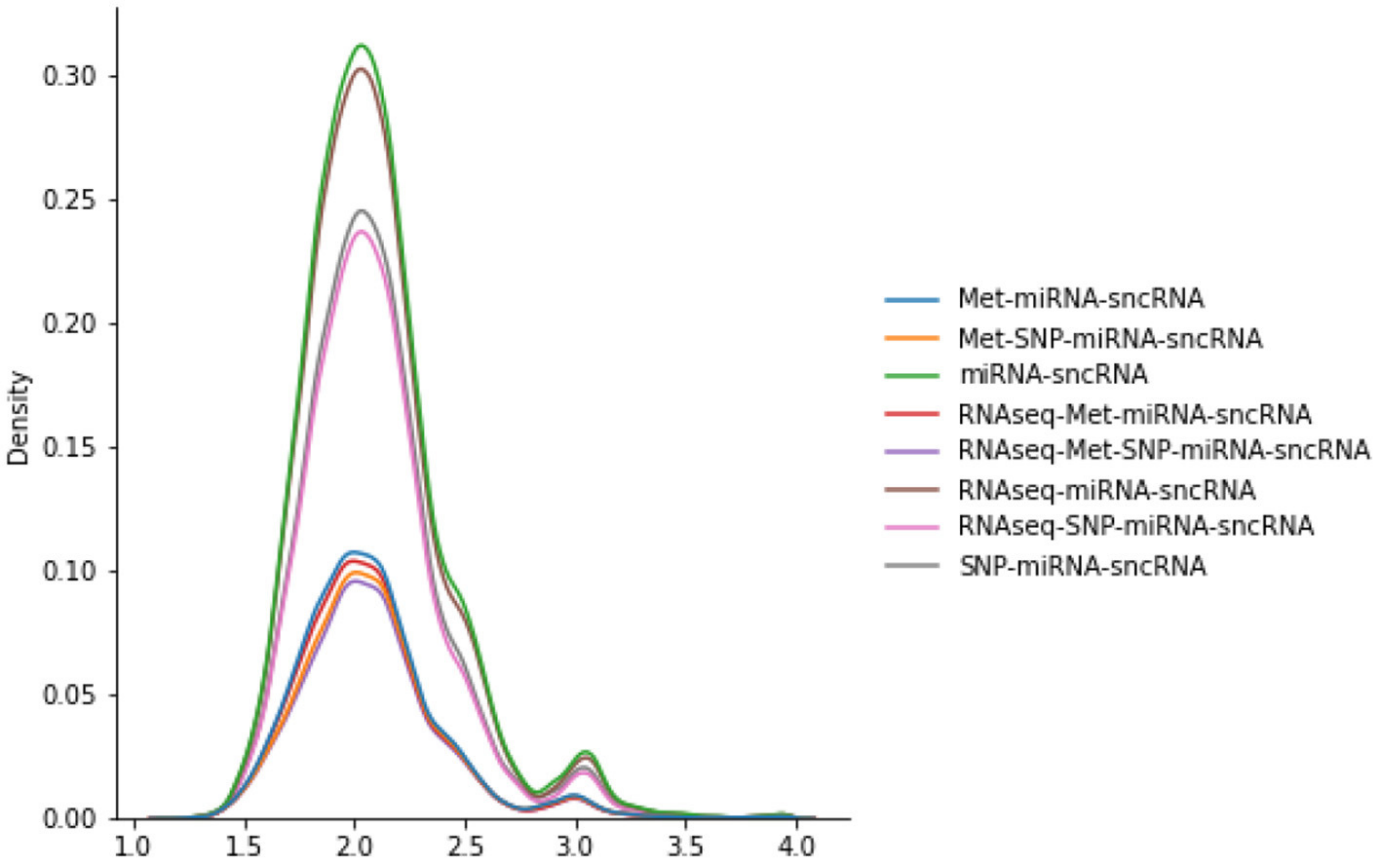
Distributions of various PSGs for DTI-fMRI data, used in the connectome encoder.

The number of scans considered in the PSG vary according to the omics combinations used in the JOIN-GCLA model. As seen in Figure 2, the PSGs have similar distributions, with most values being around 2.0 with a smaller peak around 3.0. Thus, they are not likely to explain the difference in model performances when the same imaging modalities are used, as shown in Table 4.

#### 3.11.2. Omics Population Graph Distributions

**Figure 3:**
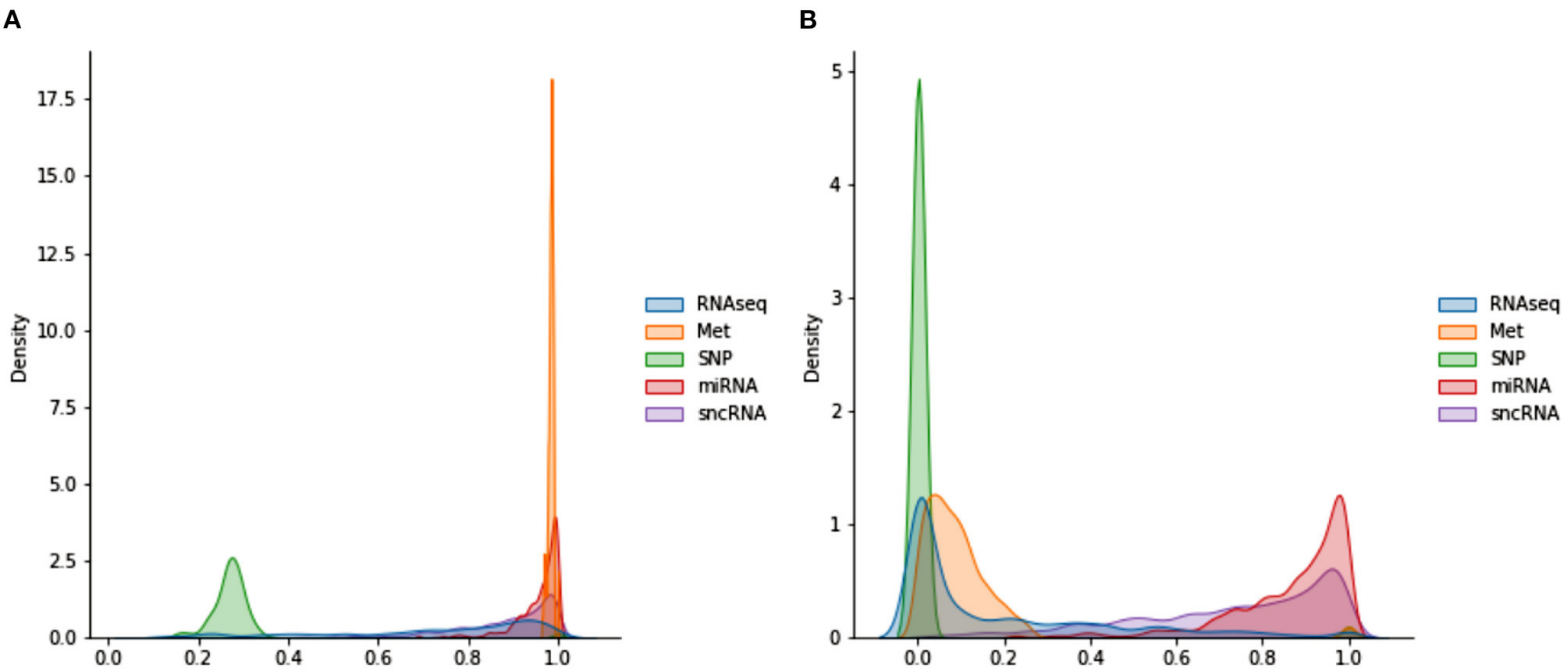
Distributions of POGs used in omics networks **(A)** before WGCNA **(B)** after WGCNA.

Figure 3 shows the distributions of POGs. These are generated by taking the lower triangular of the POG (which is symmetric) and producing kernel density plots for each omics dataset. miRNA and Met have very high values, indicating that most subjects share very similar data. While sncRNA and RNAseq has a longer left tail, SNP has a different distribution: most of the data range from 0.2 to 0.4, indicating very little similarity between subjects. When WGCNA is applied, Met and SNP clearly have very different distributions from the rest, with a majority of values being very low (below 0.3). On the other hand, miRNA and sncRNA still have most of the values above 0.6. RNAseq has many values close to 0, but also a significant amount of values spread across the range of 0 and 1.

#### 3.11.3. Attention Weights

**Figure 4:**
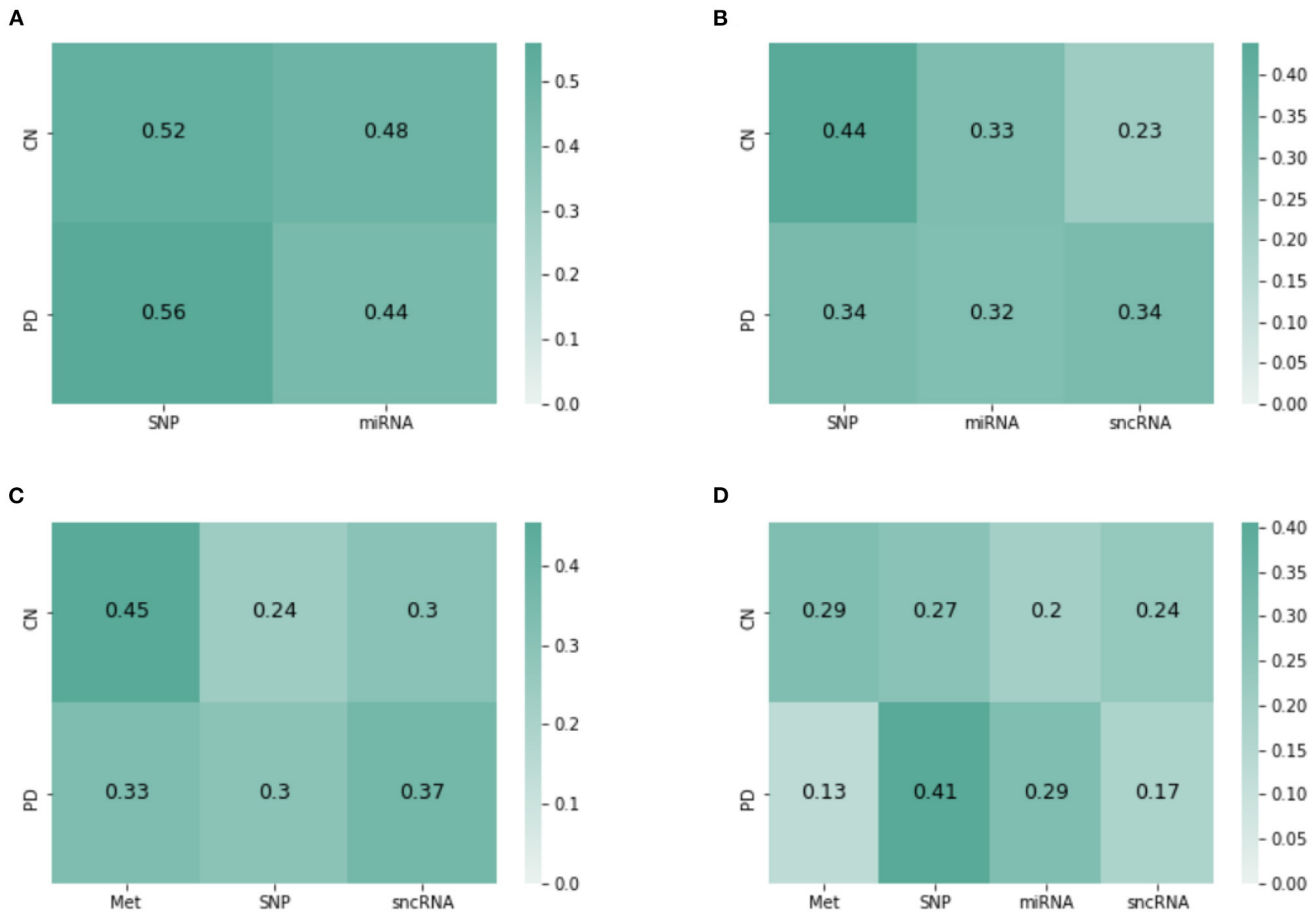
Attention matrices from JOIN-GCLA for the omics combination of **(A)** SNP-miRNA, **(B)** SNP-miRNA-sncRNA, **(C)** Met-SNP-sncRNA **(D)** Met-SNP-miRNA-sncRNA.

JOIN-GCLA provides model interpretability in the form of attention matrices with shape *N*×*L*^(4)^. In this regard, an existing method (Kazi et al., 2019a) provides a scalar value for each view. JOIN-GCLA goes further to show which view is being focused on when predicting a certain class. Figure 4A shows the attention matrix for the omics combination SNP-miRNA, which was one of the omics combinations with high MCC score. SNP has a slightly higher weight in both the cases when the model predicts HC or PD. Thus, it could be inferred that the high performance of SNP-miRNA was due to the attention mechanism's focus on SNP. Similarly, Figure 4B shows the attention matrix for SNP-miRNA-sncRNA (i.e. sncRNA is added), which had an MCC of 0.9. While the attention scores when predicting PD (the majority class) are now equally spread, the attention scores when predicting HC was heavily weighted toward SNP.

Another set of examples is presented in Figures 4C,D—with both cases having an MCC of 0.9. Met has the highest weight when predicting HC, but when miRNA was added, the attention weights are slightly more distributed between Met and SNP. Also, SNP has the highest attention score when predicting PD. It could be inferred from these attention matrices that while SNP is evidently the most important omics modality when performing disease prediction, Met contributes to the high performance too especially when predicting HC.

Overall, it can be seen that when predicting PD (majority class), the attention scores tend to focus on SNP, but it could still be equally distributed. However, when predicting HC (minority), focusing on Met (or SNP, when Met is not present) helps to improve model performance. These insights, which are more detailed than (Kazi et al., 2019a), are only possible with the use of JOIN-GCLA and our proposed attention layer.

## 4. Discussion

Overall, our results demonstrated that the combination of connectome encoder, omics networks and the customized attention layer is essential for JOIN-GCLA to work well and provide better model interpretability. From the above experiments, it is evident that our proposed architecture, JOIN-GCLA, was the best performing model. Past works have demonstrated that is it not possible to perform disease classification successfully by solely using DTI data (Prasuhn et al., 2020). Our results in Table 6 support this finding and we went further to demonstrate that disease classification can be done well if imaging and omics datasets are used simultaneously. However, datasets with both multi-modal imaging and multi-omics data are typically small. Thus, deep neural network models have to be small. JOIN-GCLA is made as lean as possible with only 1 graph convolution layer in the connectome encoder and each omics network. The number of hidden nodes is kept small as well. In the case of JOIN-GCLA, the number of parameters, as seen in Supplementary Table S4, is large in this example as the flattened correlation matrix from imaging data is used as the feature vector. However, feature vectors can be any arbitrary data of interest and thus the number of parameters could be reduced significantly, especially when dealing with smaller datasets.

It was shown in Table 8 that PSG was essential for better model performance. While JOIN-GCLA gave the best performance, there are also other important considerations such as the scalability of the model. For instance, using convolution layers instead allows for multiple connectivity matrices to be combined without a huge increase in the number of parameters as additional modalities simply increases the number of input channels. However, this comes with the limitation that connectivity matrices of the same size have to be used (i.e. same brain atlas). JOIN-GCLA is also able to merge multiple modalities *via* PSG, but if the imaging data has to be used as feature vectors (in the form of vectorised connectivity matrices), the the number of parameters increases significantly as more modalities are included, as seen in Supplementary Table S2. Thus, the model with convolution layer in the connectome encoder is most suitable for small datasets where overfitting is a concern, while JOIN-GCLA is the best choice if low-dimensional feature vectors are used.

We demonstrated the feasibility of incorporating multi-omics datasets into the model *via* the use of omics networks. As seen in Table 3, omics datasets often have a huge number of features, even more than imaging data. Thus, it is not feasible to use the entire set of omics features as feature vectors. Instead, the use of POGs allowed information from multi-omics datasets to be included into the modeling process. A population graph built from an omics dataset is used as the input graph for the graph convolution layer and fusion between the omics data and the representations of the imaging data learnt by the connectome encoder (in the form of feature vectors) happens in this graph convolution layer. Such an approach scales up well with minimal increase of parameters, as seen in Supplementary Tables S3, S4. Notably, the best model performances were obtained when 3 multi-omics datasets were used: DNA Methylation, SNP and sncRNA.

The attention layer performs a key role in combining the interim predictions from each omics network and producing a final decision. Besides performing better than baseline attention methods such as self-attention as seen in Table 9, our proposed approach ensures that an attention matrix of shape *N*×*L*^(4)^ is generated, providing greater model interpretability as seen from Figure 4. This has highlighted the relative importance of SNP and DNA Methylation in distinguishing PD patients from healthy controls.

These results were only achieved after data augmentation was introduced, as shown in Table 4. This is largely attributed to the data imbalance that exists in the PPMI dataset, with PD scans forming the majority of the data as seen in Supplementary Table S1. By comparing Table 4 with Supplementary Table S6, it is possible to observe the effects of gradually introducing more data augmentation to the DTI-fMRI dataset. When only 100 samples was added (majority class taking up 74% of the dataset), model performance did not change much as compared to the original baseline (with no augmented data, majority class takes up 93% of the dataset). But when the imbalance was further reduced by adding 200 samples (reducing the imbalance to 61%), model performance started to improve, but still significantly poorer than the performance obtained when all 339 samples were added to the dataset (resolving the imbalance, 53.3%). Since the best model performance was obtained when the data imbalance is resolved, it is evident that data augmentation is another key aspect needed to perform disease classification on the PPMI dataset successfully.

We have used the CycleGAN architecture for producing additional scans to be augmented to the original dataset. The main motivation of using CycleGAN is to overcome the limitations of the existing approaches for tackling data imbalance. As seen in Table 4 (without augmentation), class weighting applied to the loss function did not improve model performance at all, likely due to the extreme imbalance in the dataset. Undersampling is not a viable approach when dealing with small dataset, as demonstrated on an experiment in Supplementary Table S7 where the DTI dataset was undersampled—while the imbalance was well addressed (as seen in Supplementary Table S1), model performance did not improve significantly. On the other hand, oversampling on the DTI-fMRI dataset did help to improve model performance to a level similar to what was obtained from the CycleGAN-augmented dataset (comparing Table 4 with Supplementary Table S8).

While both oversampling and CycleGAN generates data that can be attributed to a specific subject (hence making it possible to link it to a genetic dataset, unlike synthetic data generation algorithms such as SMOTE and ADASYN), oversampling merely duplicates the existing dataset. CycleGAN-generated data are not just another repeated data sample in the existing dataset. However, when compared to the results obtained from oversampling, the marginal benefit introduced by the use of CycleGAN might not always justify the additional complexity added. Below, we present details on the data produced by CycleGAN to propose possible reasons for these observations.

Examples of the data generated by the CycleGAN architecture are shown in Supplementary Figure S1. Although generated scans have low mean squared errors (MSE) (approximately 0.03 when compared to actual functional connectivity matrices from the same pair ; approximately 0.5 for DTI), they do not have the same variability. On examining all the other generated matrices, it is evident that the synthetic connectomes have very slight differences and seem to capture patterns that exist across most scans, while missing out on more subtle variations that exist in functional connectivity matrices. These variations are visually stark (for fMRI), but it might not have been captured by the GAN as the overall numerical significance is not great (since the MSE achieved is rather low already). This issue is likely to be alleviated with the introduction of more data (Karras et al., 2020). Additionally, several architecture changes to the original CycleGAN were attempted to improve the variability of data generated, including adding Edge-to-edge (E2E) layers from Kawahara et al. (2017) as the first layer of the generator and discriminator and reducing the number of residual blocks (from 9 to 3) and number of filters (from 256 to 32) in the CycleGAN architecture. However, from the results in Supplementary Table S9, the best model was still the original CycleGAN architecture.

In this study, data augmentation was limited to healthy controls as the goal was to resolve the data imbalance in the PPMI dataset. Having demonstrated the feasibility of this approach, further studies could explore the use of CycleGAN to generate connectomes for various neurodegenerative disorders. Using GANs to generate connectome datasets is at a nascent stage: a recent work used GAN to generate functional connectivity matrices for schizophrenia and major depressive disorder patients (Zhao et al., 2020). Our work has extended the application of GANs on connectome datasets to multi-modal settings and the results demonstrated that using relatively large connectome datasets (~1,000 samples) to train CycleGAN is still not yet sufficient to significantly outperform oversampling as rather similar matrices are produced by the GAN. However, since CycleGAN is capable of learning from unpaired data, this is not an unsurmontable problem and future studies should consider using more data when training CycleGAN architectures to augment multi-modal connectome datasets. If obtaining more data is not feasible, oversampling presents a limited but effective approach for data augmentation.

## 5. Conclusion

We have proposed a new architecture, JOIN-GCLA, which is able to model multi-modal imaging data and multi-omics datasets simultaneously. Through the experiments, it has been demonstrated that the best performing data combination utilizes both multi-modal imaging data (DTI, fMRI) and multi-omics datasets (SNP, DNA Methylation and sncRNA). While several combinations of imaging and omics data led to very high model performance, this must be seen in the light of the small test dataset size available in the PPMI dataset. Our experiments on the PPMI dataset showed that JOIN-GCLA can work well, but this should be further tested on larger datasets that have both multi-modal imaging data and multi-omics datasets. Examples of such sources of data would be the Alzheimer's Disease Neuroimaging Initiative, UK Biobank and also future versions of PPMI, which has recently expanded its data collection with a few thousand more data samples to be expected by year 2023.

One possible area of future work is to perform decoding. Given a trained neural network model, it has been demonstrated that saliency scores can be computed to identify important features that contributed most to the model's decision (Gupta et al., 2021). While such an approach cannot be simply applied to JOIN-GCLA due to the attention layer, novel methods could be developed to weigh the saliency scores by the attention scores for each view. This could be explored as a follow-up work after this paper.

Another direction for further research on combining neuroimaging and omics datasets is the use of transformers. While originally proposed for natural language processing (Vaswani et al., 2017), it has been demonstrated to work on images too (Dosovitskiy et al., 2020), motivating recent works on using transformer-based architectures for multi-modal settings (Hu and Singh, 2021; Kim et al., 2021). One limitation of such models is their reliance on pre-training from large datasets (Dosovitskiy et al., 2020). Modifying transformers to work on small datasets is still an open area of research (Lee et al., 2021). This could explain the paucity of works on using transformers for neuroimaging datasets (especially on connectivity matrices). Recent works on the use of transformers utilizes raw fMRI signals (Nguyen et al., 2020; Malkiel et al., 2021). Notably, one of the key findings in Malkiel et al. (2021) is the need for pre-training for best model performance. Addressing this issue for connectome datasets could be possible with the use of larger datasets such as UK Biobank.

While this paper focuses on PD classification using multi-modal imaging data (DTI, fMRI) and multi-omics data (miRNA, DNA methylation, RNAseq, sncRNA, SNP), JOIN-GCLA can be easily extended to other diseases, omics modalities and imaging modalities too. For instance, diseases such as ADHD could benefit from the use of multi-modal imaging and multi-omics data (Klein et al., 2017) and the problem of limited multi-modal data could be addressed by using CycleGAN to generate more data. However, our results suggest that such approaches will need large amounts of data (more than 1,000 data points) to train the CycleGAN architecture.

In sum, the JOIN-GCLA architecture makes it possible to analyse multi-modal imaging data along with multi-omics datasets. Our proposed architecture alleviates the issue of high dimensionality of imaging and omics data by incorporating them in graph convolution layers in the form of PSG and POG, respectively. This enables multi-scale analysis, incorporating both macro-scale imaging data with micro-scale genomics analysis, to be conducted. The greater interpretability provided by JOIN-GCLA's attention matrices gives greater insight into the relative importance of the omics datasets taken into consideration, potentially revealing more novel insights for complex neurodegenerative diseases in future studies.

## Data Availability Statement

The original contributions presented in the study are publicly available. This data can be found here: Dataset provided by PPMI can be found in https://www.ppmi-info.org/. Information about HCP S1200 are presented in https://www.humanconnectome.org/study/hcp-young-adult/document/1200-subjects-data-release and the dataset can be downloaded from https://db.humanconnectome.org/. Download links for the AOMIC dataset are provided at https://nilab-uva.github.io/AOMIC.github.io/.

## Author Contributions

All authors listed have made a substantial, direct, and intellectual contribution to the work and approved it for publication.

## Funding

This work was partially supported by AcRF Tier-1 grant RG116/19 and AcRF Tier-2 grant MOE T2EP20121-0003 of Ministry of Education, Singapore.

## Conflict of Interest

The authors declare that the research was conducted in the absence of any commercial or financial relationships that could be construed as a potential conflict of interest.

## Publisher's Note

All claims expressed in this article are solely those of the authors and do not necessarily represent those of their affiliated organizations, or those of the publisher, the editors and the reviewers. Any product that may be evaluated in this article, or claim that may be made by its manufacturer, is not guaranteed or endorsed by the publisher.

## Footnotes

^1^Data augmentation was done by using the trained CycleGAN model to generate functional connectivity matrices only as the PPMI dataset has too few fMRI scans to generate structural connectivity matrices from. Instead, the DTI dataset is used to demonstrate effects of undersampling, as shown in Supplementary Table S7.
