# Supplementary material for: Combining Neuroimaging and Omics Datasets for Disease Classification Using Graph Neural Networks
Source: Front Neurosci. 2022 May 23;16:866666. doi: 10.3389/fnins.2022.866666 (PMC9168232; doi:10.3389/fnins.2022.866666)
Supplement: Supplementary file 1 [file Data_Sheet_1.PDF]

Supplementary Material

1 DATASET INFORMATION

Table S1. Additional information about each omics combination. n_ratio = Proportion of majority class (PD) in test set. n = number of scans in test set. Under = Undersampling was performed.

	DTI (Original)		DTI (Under)		fMRI		DTI-fMRI	
Omics	n_ratio	n	n_ratio	n	n_ratio	n	n_ratio	n
None	0.80	259.8	0.48	98.0	0.92	68.1	0.93	114.8
Met	0.81	189.9	0.51	73.0	0.89	50.8	0.90	88.9
SNP	0.80	259.8	0.48	98.0	0.92	68.1	0.93	114.8
miRNA	0.81	248.9	0.47	91.0	0.95	55.7	0.96	99.4
sncRNA	0.81	248.9	0.47	91.0	0.95	55.7	0.96	99.4
RNAseq	0.80	289.7	0.48	108.7	0.91	72.5	0.93	128.5
RNAseq-Met	0.82	190.2	0.51	70.9	0.89	49.6	0.90	88.9
RNAseq-SNP	0.81	261.1	0.48	97.2	0.91	64.1	0.93	114.8
RNAseq-miRNA	0.81	247.3	0.48	89.2	0.94	53.5	0.96	99.4
RNAseq-sncRNA	0.81	247.3	0.48	89.2	0.94	53.5	0.96	99.4
Met-SNP	0.82	178.3	0.51	66.4	0.89	48.2	0.90	79.4
Met-miRNA	0.83	157.6	0.52	55.9	0.91	32.2	0.97	66.8
Met-sncRNA	0.83	157.6	0.52	55.9	0.91	32.2	0.97	66.8
SNP-miRNA	0.82	215.9	0.50	77.5	0.94	50.4	0.98	94.2
SNP-sncRNA	0.82	215.9	0.50	77.5	0.94	50.4	0.98	94.2
miRNA-sncRNA	0.81	248.9	0.47	91.0	0.95	55.7	0.96	99.4
RNAseq-Met-SNP	0.81	169.6	0.51	65.3	0.88	47.6	0.90	79.4
RNAseq-Met-miRNA	0.82	155.4	0.51	55.2	0.91	32.8	0.97	66.8
RNAseq-Met-sncRNA	0.82	155.4	0.51	55.2	0.91	32.8	0.97	66.8
RNAseq-SNP-miRNA	0.82	216.9	0.50	76.2	0.94	48.9	0.98	94.2
RNAseq-SNP-sncRNA	0.82	216.9	0.50	76.2	0.94	48.9	0.98	94.2
RNAseq-miRNA-sncRNA	0.81	247.3	0.48	89.2	0.94	53.5	0.96	99.4
Met-SNP-miRNA	0.81	147.7	0.49	53.7	0.91	31.8	0.97	56.9
Met-SNP-sncRNA	0.81	147.7	0.49	53.7	0.91	31.8	0.97	56.9
Met-miRNA-sncRNA	0.83	157.6	0.52	55.9	0.91	32.2	0.97	66.8
SNP-miRNA-sncRNA	0.82	215.9	0.50	77.5	0.94	50.4	0.98	94.2
RNAseq-Met-SNP-miRNA	0.80	142.9	0.47	53.6	0.90	31.4	0.97	56.9
RNAseq-Met-SNP-sncRNA	0.80	142.9	0.47	53.6	0.90	31.4	0.97	56.9
RNAseq-Met-miRNA-sncRNA	0.82	155.4	0.51	55.2	0.91	32.8	0.97	66.8
RNAseq-SNP-miRNA-sncRNA	0.82	216.9	0.50	76.2	0.94	48.9	0.98	94.2
Met-SNP-miRNA-sncRNA	0.81	147.7	0.49	53.7	0.91	31.8	0.97	56.9
RNAseq-Met-SNP-miRNA-sncRNA	0.80	142.9	0.47	53.6	0.90	31.4	0.97	56.9

2 DATASET PRE-PROCESSING

2.1 Parkinson's Progression Markers Initiative (PPMI)

2.1.1 DTI pre-processing

These results have been obtained using the `dwi-preprocessing` pipeline of Clinica (Routier et al., 2021). For each subject, all raw diffusion weighted imaging (DWI) volumes were rigidly registered (6 degrees of freedom (dof)) to the reference b_0 image (DWI volume with no diffusion sensitization) to correct for head motion. The diffusion weighting directions were appropriately updated (Leemans and Jones, 2009). An affine registration (12 dof) was then performed between each DWI volume and the reference b_0 to correct for eddy current distortions. These registrations were done using the FSL flirt tool (`www.fmrib.ox.ac.uk/fsl`). To correct for echo-planar imaging (EPI) induced susceptibility artifacts, the skull-stripped b_0 images were registered to the T1-weighted image using the ANTs SyN registration algorithm (Avants et al., 2008; Leow et al., 2007). The resulting deformation fields were then applied to the DWI volumes to align them with the T1 image. Finally, the DWI volumes were corrected for nonuniform intensity using the ANTs N4 bias correction algorithm (Tustison et al., 2010). A single multiplicative bias field from the reference b_0 image was estimated, as suggested in (Jeurissen et al., 2014).

`BedpostX GPU` (Hernández et al., 2013) was used to fit a probabilistic diffusion model and probabilistic tractography was performed via `ProbtrackX GPU` (Hernandez-Fernandez et al., 2019) to generate structural connectivity matrices. Although the GPU-accelerated version of the tools were used, pre-processing still took a significant amount of time and thus the parameter `nsamples` was set as 1000. Adjusting this value higher or lower produced very similar streamline counts (after normalising it by dividing by `nsamples`). The remaining parameters used are based on the default settings (e.g. `nsteps` = 2000). The AAL atlas was used to define 116 regions of interest and the `--network` flag was used to generate the raw structural connectivity matrix.

2.1.2 fMRI pre-processing

The following boilerplate text was generated from the fMRIPrep pipeline (Esteban et al., 2019), released under a CC0 license.

For each fMRI scan for a subject, the following preprocessing was performed. First, a reference volume and its skull-stripped version were generated and the BOLD reference was co-registered to the T1w reference using `bbregister` (FreeSurfer). Head-motion parameters with respect to the BOLD reference (transformation matrices, and six corresponding rotation and translation parameters) are estimated before any spatiotemporal filtering using `mcflirt` (Jenkinson et al., 2002). BOLD runs were slice-time corrected using `3dTshift` from AFNI (Cox and Hyde, 1997). The BOLD time-series were resampled onto their original, native space by applying a single, composite transform to correct for head-motion and susceptibility distortions.The BOLD time-series were resampled to `MNI152NLin2009cAsym` standard space, generating a preprocessed BOLD run in MNI152NLin2009cAsym space. Principal components are estimated after high-pass filtering the preprocessed BOLD time-series. The head-motion estimates calculated in the correction step were also placed within the corresponding confounds file. The BOLD time-series, were resampled to *fsaverage5* surfaces with *a single interpolation step* by composing all the pertinent transformations (i.e. head-motion transform matrices, susceptibility distortion correction when available, and co-registrations to anatomical and template spaces). Gridded (volumetric) resamplings were performed using antsApplyTransforms (ANTs), configured with Lanczos interpolation to minimize the smoothing effects of other kernels (Lanczos, 1964). Non-gridded (surface) resamplings were performed using `mri_vol2surf` (FreeSurfer).

2.1.3 Multi-omics pre-processing

Multi-omics data are provided by PPMI (`https://www.ppmi-info.org/`). Whole-transcriptome RNA Sequencing (RNA-Seq) data was sequenced on an Illumina NovaSeq6000, and quality control was conducted to generate read counts and transcripts per million (TPM) measurement. TPM data is an abundance estimate of genes acquired by Salmon calculated by normalizing the counts by gene length. Since gene length varies significantly and more reads are generally mapped to longer transcripts, the expression level is biased when measuring with raw read counts (Zhao et al., 2020). Therefore, in this research, we used TPM data for downstream analysis to alleviate this bias. Small RNA Sequencing data, including small non-coding RNA (sncRNA) and micro RNA (miRNA), was sequenced on a same platform with RNA-Seq, and sophisticated quality control was conducted to ensure all kinds of small RNA were properly measured. Their expression values were provided in format of read counts, reads per million (RPM) and reads per million mapped to miRNA (RPMMM) specially for miRNA. Due to a similar reason, we used RPMMM data and RPM data to quantify miRNA and scnRNA, respectively.

DNA Methylation data of around 850,000 CpG probes was measured by Illumina Human MethylationEPIC BeadChip protocol and Illumina iScan System per manufacturer's protocol. Quality control was conducted to remove samples that failed in sex discrepancy and p-value detection test. Then functional normalization was performed to remove probes with p-value over 0.01.

Single Nucleotide Polymorphism (SNP) genotyping was performed on the whole-blood extracted DNA samples by Illumina Human Omni Express Exome+ v1.3NeuroX array. Quality control was conducted to remove unqualified samples according to sex discrepancy determined by X chromosome heterogeneity.

A pre-processing routine involving simple noise removal and Wilcoxon signed rank test is performed for each omics dataset. During simple noise removal procedure, any missing data are filled with zero and multi-omics entities are eliminated if their values remain to be zero along no less than 90% of the subjects. Afterwards, Wilcoxon signed rank test (Woolson, 2007) is applied for identifying the relevant features ($p < 0.001$) with PD diagnosis trait in multi-omics datasets.

2.2 Human Connectome Project (HCP)

Structural and functional brain imaging data from the HCP S1200 dataset was used to generate structural and functional connectivity matrices, which are subsequently used to train the CycleGAN model.

2.2.1 DTI pre-processing

Pre-processed DTI data downloaded from HCP was used in this study. The HCP diffusion pipeline involves intensity normalisation of the b_0 image across runs, removal of echo planar imaging and eddy current induced distortion as well as motion and gradient-nonlinearity correction (Andersson and Sotiropoulos, 2016). The diffusion images were then registered to the structural images and brought into the 1.25mm structural space before brain extraction was performed. This set of pre-processed DTI images was then used to generate the structural connectivity matrices, via the same steps outlined in Section 2.1.1.

2.2.2 fMRI pre-processing

Pre-processed resting state fMRI (rs-fMRI) data downloaded from HCP was used in this study. In the HCP fMRIVolume preprocessing pipeline, gradient nonlinearity distortions are first corrected before motion correction was performed via FSL's FLIRT with 6 degrees of freedom. Echo planar distortion is corrected via FSL's `topup` tool. A non-linear registration is then performed to bring the fMRI images to the MNI standard space.

To generate the functional connectivity matrix, the same steps used on the PPMI pre-processed were performed: AAL atlas was used to delineate 116 ROIs and the time series of voxels within 2.5mm of the ROI were averaged. Pearson correlation coefficient is then computed on these 116 mean time series to arrive at a symmetric functional connectivity matrix for each scan.

Subjects in the HCP rs-fMRI dataset often took multiple scans (ranging from 1 to 4). Each scan lasted for 15 minutes and subjects were told to keep their eyes open while keeping their gaze fixated at a screen with a white cross on a dark background (Smith et al., 2013). For the subjects with multiple scans, their connectivity matrices was averaged - Fisher z-transform was first performed before the averaging and after the averaging, the inverse Fisher z-transformation was done.

2.3 Amsterdam Open MRI Collection (AOMIC)

Structural and functional brain imaging data from the AOMIC dataset (Snoek et al., 2021) was used to generate structural and functional connectivity matrices, which are subsequently used to tune the CycleGAN model. Specifically, the PIOP1 data subset was used as validation set, while the PIOP2 data subset was used as test set.

2.3.1 DTI pre-processing

MRtrix3 was used to perform denoising, removal of Gibbs ringing artefacts, eddy current correction and motion correction from the DWI images. Similar to what was done for PPMI DWI images as described in Section 2.1.1, the GPU versions of BedpostX and ProbtrackX from FSL was used to generate the structural connectivity matrices.

2.3.2 fMRI pre-processing

Pre-processed rs-fMRI data downloaded from AOMIC was used in this study. The fMRIprep tool was used to perform pre-processing. Notably, slice timing correction was not performed and motion correction was done via `mcflirt`. Distortion correction was done without fieldmaps in a similar approach as what was done in the PPMI dataset (co-registeration to a T1w image via ANTs). The images were then registered to the MNI standard space. Generation of the functional connectivity matrices followed the same steps as mentioned in Section 2.2.2.

Each subject only has 1 scan (lasting 6 minutes for PIOP1 and 8 minutes for PIOP2) and the scanning conditions were similar to the HCP dataset - subjects were told to keep their eyes open and gaze fixed at a white cross on a grey background. Notable difference includes the dark background used in HCP (vs grey background in AOMIC) and subjects in HCP were told to not think about anything in particular while subjects in AOMIC were told to let their thoughts run freely. Notable differences between PIOP1 and PIOP2 includes the difference in acquistion (the former was recorded with a multiband acqusition with TR of 750ms while the latter was recorded with a sequential acqusition with TR of 2000ms).

3 ADDITIONAL RESULTS

3.1 Comparison of model parameters

Table S2. Comparison of the number of parameters in each model, for the example of SNP. One = one imaging modality. Both = DTI + fMRI.

Model	No omics	Single omics
Params (One)	107042	107314
Params (Both)	213702	214034

Table S3. Comparison of the number of parameters in each model, for the example of Met-miRNA.

Model	JOIN-GCLA	Fully-connected layer	Convolution layer	Self attention
Params (Both)	214330	214058	34042	214330

Table S4. Comparison of the number of parameters in each model, for the example of RNAseq-Met-SNP-miRNA-sncRNA.

Model	JOIN-GCLA	Fully-connected layer	Convolution layer	Self attention
Params (Both)	215146	214874	34858	215146

3.2 Additional experiments for connectome encoder

Table S5. Model performance for various sizes of convolution layers in connectome encoder. Larger = 40 hidden neurons and filters, original = 16.

	Original layers		Larger layers	
Omics	Accuracy	MCC	Accuracy	MCC
Model 3	95.59 ± 0.09	0.57 ± 0.46	92.12 ± 0.09	0.43 ± 0.31
Model 4	88.62 ± 0.23	0.26 ± 0.34	95.56 ± 0.05	0.39 ± 0.45
Model 5	72.86 ± 0.32	0.23 ± 0.30	94.94 ± 0.09	0.65 ± 0.41

3.3 Model performance decreases sharply when less data is augmented

Table S6. Model performance when less data samples were added to DTI-fMRI training set for the JOIN-GCLA model.

	100 samples added		200 samples added		339 samples added (full)	
Omics	Accuracy	MCC	Accuracy	MCC	Accuracy	MCC
Model 3	82.23 ± 0.12	0.28 ± 0.28	97.96 ± 0.02	0.40 ± 0.51	100.0 ± 0.00	1.00 ± 0.00
Model 4	89.81 ± 0.21	0.06 ± 0.15	97.23 ± 0.02	0.25 ± 0.41	93.21 ± 0.21	0.91 ± 0.29
Model 5	96.78 ± 0.01	0.19 ± 0.31	86.71 ± 0.29	0.24 ± 0.41	89.67 ± 0.30	0.73 ± 0.44

3.4 Undersampling is not feasible for small and very imbalanced datasets

Table S7. Comparison of model performance between original and undersampled versions of DTI dataset on the JOIN-GCLA model.

	Without undersampling		With undersampling	
Omics	Accuracy	MCC	Accuracy	MCC
RNAseq-Met	77.67 ± 0.12	0.27 ± 0.21	68.04 ± 0.06	0.38 ± 0.12
RNAseq-SNP	80.31 ± 0.04	0.24 ± 0.15	60.03 ± 0.04	0.24 ± 0.06
RNAseq-miRNA	76.52 ± 0.07	0.14 ± 0.14	59.80 ± 0.06	0.24 ± 0.12
RNAseq-sncRNA	76.74 ± 0.07	0.14 ± 0.12	59.22 ± 0.06	0.19 ± 0.15
Met-SNP	79.83 ± 0.09	0.33 ± 0.19	60.21 ± 0.05	0.24 ± 0.15
Met-miRNA	85.54 ± 0.03	0.32 ± 0.28	54.44 ± 0.06	0.07 ± 0.14
Met-sncRNA	84.63 ± 0.03	0.28 ± 0.25	61.89 ± 0.07	0.26 ± 0.18
SNP-miRNA	82.28 ± 0.05	0.34 ± 0.15	59.87 ± 0.07	0.24 ± 0.18
SNP-sncRNA	81.35 ± 0.06	0.32 ± 0.17	64.26 ± 0.07	0.29 ± 0.14
miRNA-sncRNA	80.96 ± 0.01	0.05 ± 0.10	59.59 ± 0.05	0.20 ± 0.13
RNAseq-Met-SNP	76.87 ± 0.16	0.22 ± 0.19	65.03 ± 0.06	0.32 ± 0.16
RNAseq-Met-miRNA	83.48 ± 0.08	0.43 ± 0.22	69.17 ± 0.08	0.43 ± 0.14
RNAseq-Met-sncRNA	85.27 ± 0.04	0.43 ± 0.20	70.64 ± 0.08	0.44 ± 0.16
RNAseq-SNP-miRNA	81.01 ± 0.06	0.27 ± 0.15	60.24 ± 0.06	0.21 ± 0.12
RNAseq-SNP-sncRNA	81.53 ± 0.05	0.25 ± 0.14	63.67 ± 0.09	0.28 ± 0.19
RNAseq-miRNA-sncRNA	80.84 ± 0.02	0.07 ± 0.10	59.01 ± 0.06	0.22 ± 0.11
Met-SNP-miRNA	83.74 ± 0.02	0.31 ± 0.18	61.45 ± 0.08	0.26 ± 0.21
Met-SNP-sncRNA	82.48 ± 0.05	0.34 ± 0.14	60.59 ± 0.09	0.21 ± 0.21
Met-miRNA-sncRNA	83.43 ± 0.04	0.29 ± 0.20	62.03 ± 0.09	0.27 ± 0.21
SNP-miRNA-sncRNA	81.39 ± 0.06	0.29 ± 0.18	60.34 ± 0.06	0.23 ± 0.14
RNAseq-Met-SNP-miRNA	80.53 ± 0.07	0.30 ± 0.21	65.61 ± 0.08	0.32 ± 0.19
RNAseq-Met-SNP-sncRNA	79.86 ± 0.08	0.30 ± 0.20	62.5 ± 0.09	0.28 ± 0.17
RNAseq-Met-miRNA-sncRNA	81.88 ± 0.10	0.36 ± 0.19	71.16 ± 0.07	0.45 ± 0.14
RNAseq-SNP-miRNA-sncRNA	82.85 ± 0.05	0.28 ± 0.16	57.80 ± 0.06	0.19 ± 0.12
Met-SNP-miRNA-sncRNA	81.55 ± 0.05	0.27 ± 0.21	62.20 ± 0.11	0.26 ± 0.24
RNAseq-Met-SNP-miRNA-sncRNA	79.70 ± 0.06	0.22 ± 0.22	67.00 ± 0.08	0.36 ± 0.17

3.5 Comparison between data augmentation via CycleGAN and oversampling

Table S8. Model performance when oversampling is performed on DTI-fMRI training set, compared to using CycleGAN for data augmentation

	CycleGAN		Oversampling	
Omics	Accuracy	MCC	Accuracy	MCC
Model 3	100.0 ± 0.00	1.00 ± 0.00	99.21 ± 0.02	0.86 ± 0.33
Model 4	93.21 ± 0.21	0.91 ± 0.29	99.68 ± 0.01	0.90 ± 0.32
Model 5	89.67 ± 0.30	0.73 ± 0.44	83.63 ± 0.34	0.61 ± 0.50

3.6 Ablation study of the CycleGAN architecture

Table S9. Model performance for various changes to the original CycleGAN architecture. MSE = mean squared error.

	fMRI		DTI	
	MSE at epoch 20	MSE at epoch 1000	MSE at epoch 20	MSE at epoch 1000
CycleGAN	0.053	0.042	0.71	0.59
CycleGAN_E2E	0.047	0.056	0.65	0.76
CycleGAN_small	0.053	0.045	0.75	0.65

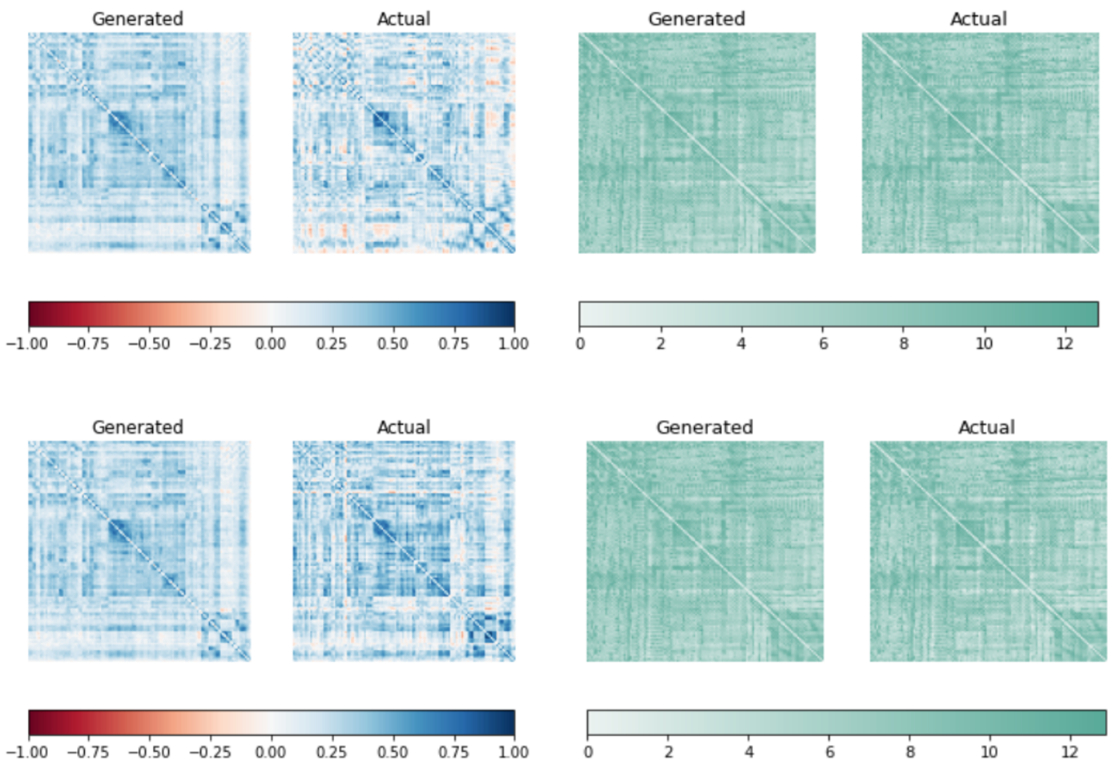

Figure S1. Example of functional and structural connectomes generated by the CycleGAN architecture. Values in functional connectomes range from -1 to 1 as they represent Pearson correlation coefficient between pairs of regions of interests (ROI). Values in structural connectome represent the number of streamlines that connects pairs of ROIs, after log transformation.

REFERENCES

Andersson, J. L. and Sotiropoulos, S. N. (2016). An integrated approach to correction for off-resonance effects and subject movement in diffusion mr imaging. *Neuroimage* 125, 1063–1078

Avants, B. B., Epstein, C. L., Grossman, M., and Gee, J. C. (2008). Symmetric diffeomorphic image registration with cross-correlation: evaluating automated labeling of elderly and neurodegenerative brain. *Medical image analysis* 12, 26–41

Cox, R. W. and Hyde, J. S. (1997). Software tools for analysis and visualization of fmri data. *NMR in Biomedicine* 10, 171–178. doi:10.1002/(SICI)1099-1492(199706/08)10:4/5⟨171::AID-NBM453⟩3.0.CO;2-L

Esteban, O., Markiewicz, C. J., Blair, R. W., Moodie, C. A., Isik, A. I., Erramuzpe, A., et al. (2019). fmriprep: a robust preprocessing pipeline for functional mri. *Nature methods* 16, 111–116

Hernández, M., Guerrero, G. D., Cecilia, J. M., García, J. M., Inuggi, A., Jbabdi, S., et al. (2013). Accelerating fibre orientation estimation from diffusion weighted magnetic resonance imaging using gpus. *PloS one* 8, e61892

Hernandez-Fernandez, M., Reguly, I., Jbabdi, S., Giles, M., Smith, S., and Sotiropoulos, S. N. (2019). Using gpus to accelerate computational diffusion mri: From microstructure estimation to tractography and connectomes. *Neuroimage* 188, 598–615

Jenkinson, M., Bannister, P., Brady, M., and Smith, S. (2002). Improved optimization for the robust and accurate linear registration and motion correction of brain images. *NeuroImage* 17, 825–841. doi:10.1006/nimg.2002.1132

Jeurissen, B., Tournier, J.-D., Dhollander, T., Connelly, A., and Sijbers, J. (2014). Multi-tissue constrained spherical deconvolution for improved analysis of multi-shell diffusion mri data. *NeuroImage* 103, 411–426

Lanczos, C. (1964). Evaluation of noisy data. *Journal of the Society for Industrial and Applied Mathematics Series B Numerical Analysis* 1, 76–85. doi:10.1137/0701007

Leemans, A. and Jones, D. K. (2009). The b-matrix must be rotated when correcting for subject motion in dti data. *Magnetic Resonance in Medicine: An Official Journal of the International Society for Magnetic Resonance in Medicine* 61, 1336–1349

Leow, A. D., Yanovsky, I., Chiang, M.-C., Lee, A. D., Klunder, A. D., Lu, A., et al. (2007). Statistical properties of jacobian maps and the realization of unbiased large-deformation nonlinear image registration. *IEEE transactions on medical imaging* 26, 822–832

Routier, A., Burgos, N., Díaz, M., Bacci, M., Bottani, S., El-Rifai, O., et al. (2021). Clinica: an open-source software platform for reproducible clinical neuroscience studies. *Frontiers in Neuroinformatics* 15

Smith, S. M., Beckmann, C. F., Andersson, J., Auerbach, E. J., Bijsterbosch, J., Douaud, G., et al. (2013). Resting-state fmri in the human connectome project. *Neuroimage* 80, 144–168

Snoek, L., van der Miesen, M. M., Beemsterboer, T., van der Leij, A., Eigenhuis, A., and Scholte, H. S. (2021). The amsterdam open mri collection, a set of multimodal mri datasets for individual difference analyses. *Scientific data* 8, 1–23

Tustison, N. J., Avants, B. B., Cook, P. A., Zheng, Y., Egan, A., Yushkevich, P. A., et al. (2010). N4itk: improved n3 bias correction. *IEEE transactions on medical imaging* 29, 1310–1320

Woolson, R. F. (2007). Wilcoxon signed-rank test. *Wiley encyclopedia of clinical trials* , 1–3

Zhao, S., Ye, Z., and Stanton, R. (2020). Misuse of rpkm or tpm normalization when comparing across samples and sequencing protocols. *Rna* 26, 903–909
